# Hospital and Institutional News

**Published:** 1916-10-21

**Authors:** 


### HOSPITAL AND INSTITUTIONAL NEWS.

### THE EMPRESS EUGENIE'S HOSPITAL FOR WOUNDED OFFICERS.

Already in her ninety-first year, that venerable lady, the Empress Eugénie, has converted to use as a war hospital a large wing of the mansion on Farnborough Hill which she has made her home. The hospital was arranged under the Empress's personal supervision, and has been equipped with all the requisites for its purpose. Each officer has his own bedroom, and there are common rooms for meals, reading, and recreation. Their hostess calls every morning to inquire after the health of her guests, and she is always concerned to promote their comfort and amusement. Revolving huts have been put up in the fine park of two hundred acres in which the house stands, so that officers can lie out of doors and yet obtain the greatest amount of sunshine and the least amount of wind at any time of the day or year. One hundred and fifty officers have already passed through this splendid auxiliary hospital; and fortunate indeed are they who thus find themselves the guests of the great lady whom England has been proud to honour these forty-six years, ever since the previous occasion on which the hordes of barbarism swept over France. The tragedy of her life-story none can efface, but there will be a peculiar appropriateness in the final downfall of Germany within the lifetime of the Empress Eugénie, and it is understood that she follows the course of the war with the keenest interest.

### OPENING OF THE NEW RUSSIAN HOSPITAL.

By the generosity of M. Mouravieff-Apostol, Chamberlain of the Russian Court, a Russian Hospital for British Officers has been opened in South Audley Street, and will be maintained by the same donor. On October 17 the hospital was formally opened by the Prime Minister, after the dedication and blessing according to the rites of the Orthodox Church by the Rev. Father Smirnoff, Chaplain to the Russian Embassy. The hospital will be under the direct patronage of the Dowager Empress of Russia, and will be named St. Mary's Hospital, after her patron saint. Sir Berkeley and Lady Sheffield have lent their house in South Audley Street during the war, but after peace is declared it is intended to remove to permanent quarters elsewhere in London as a civil Russian hospital. Thirty beds are now ready, and it is hoped that the number will soon be brought up to fifty. Mme. Mouravieff-Apostol will herself supervise the hospital as commandant, but the staff will be entirely English, including several doctors who have already spent some months in Russia on the staff of the Anglo-Russian Hospital. Dr. Gould May is in medical charge.

### THE SMALL MILITARY HOSPITAL.

The need for further hospital accommodation for wounded men is not yet fully met, and certain areas and counties are confronted with the problem of supplying a larger quota of beds than has been previously required of them. It is pointed out that the military authorities prefer to have not fewer than fifty beds in the small hospitals, and the effect of this not unnatural preference is the moving of some of the existing small hospitals into larger buildings, so that a multiplication of hospitals with twenty beds or so may be avoided. As a result of this administrative change, only those small hospitals which throughout have not availed themselves of the Government grant are likely to remain in their old quarters, and it says much for the private generosity of Englishmen on the one hand, and for the English hatred of official regulations on the other, that such privately run hospitals still continue in the third year of war. In this conviction, a supporter of one of these institutions lately made a characteristic remark in our hearing. Asked for what national ideal the English in particular were fighting, he replied, with an emphasis as quaint as it was transparently sincere: "We are fighting for the old English inefficiency—against the drill-sergeant, the State official, organisation, and rules." The small hospital in which he is interested contains twenty odd beds, and will *not* be one of those to move!

### CAVELL MEMORIAL AT SHOREDITCH INFIRMARY.

On October 12, the first anniversary of her death, a memorial to Edith Cavell was unveiled at the Shoreditch Infirmary, where she was for a few years the assistant matron. The memorial is a painting in three panels symbolising Faith, Hope, and Charity. A letter was read from Mr. Walter Long, President of the Local Government Board, drawing attention to the honour conferred on the Poor-Law Nursing Service by the fact that Miss Cavell had once been in its ranks. Dr. Addison, M.P., attended the ceremony and delivered an address, eulogising the memory of Miss Cavell, and denouncing the spirit of faction as contrasted with that of devotion exemplified by her career and by all those who were making sacrifices at home or abroad in the cause for which the Allies are fighting. At Brighton the anniversary was marked by a procession through the streets, followed by a largely attended meeting under the auspices of the British Empire Union and presided over by the Mayor.

### THE SCANDAL OF THE INDIAN HOSPITALS.

The persistence of the *Morning Post* in tracking down and exposing the lamentable deficiencies of the Army hospital system in India, and the officially admitted negligence of the authorities there, is bringing to light scandals for which those responsible ought to be brought very promptly and seriously to book. The latest revelation concerns a hospital the name of which is not given; the report is a first-hand communication from a nurse who is working in it. Old and rotten beds, no punkahs, defective mosquito curtaining hopelessly beyond repair and quite ineffective, abundance of bugs, poor water supply, absence of a dependable supply of ice, bad food, and not nearly enough nursing appliances are some of the principal counts of the indictment. Now everyone of these disgraceful hardships for the wounded is avoidable; and steps should be taken not only to remedy them at once, but also to fix responsibility on every inspecting officer who has visited this hospital in the last two years. If half-a-dozen colonels and surgeon-generals were disgraced, we are confident these abominable conditions in Indian hospitals would soon cease to be. For the conduct of the servants, who are stated to steal the soldiers' kits, for which the latter are mulcted by stoppages of their pay, the commandant of the hospital must be held responsible, and should be firmly dealt with. Until the Government of India makes up its mind to insist on efficiency in its medical departments, scandals such as these will increase and multiply.

### HOSPITAL FINANCE IN NEW ZEALAND.

It will be remembered that the system under which the hospitals of New Zealand are financed includes a provision, under statute, whereby the Government grant is partly based upon the sum levied by the hospital boards from contributing local authorities. As the latter amount depends upon the rateable value per head of the population of the district served by a hospital, there is always the possibility of the ratepayer of one hospital area finding himself at a disadvantage as compared with the ratepayer of another area. A low State subsidy, it follows, means that the general taxpayer is relieved at the expense of the local taxpayer. These points were brought out when the representatives of local bodies who help to finance the Wellington Public Hospital waited on the Minister of Public Health to ask, amongst other things, for a larger subsidy for their district hospital. It appears that the rate of subsidy in the pound for the Wellington Board was 16s. 3d., against 18s. 3d. for the Chichester Hospital Board, 19s. 3d. for Auckland, and £1 1s. 9d. for Otago. One of the points brought out by the deputation was that although the people using the local hospital came from all parts of New Zealand the local ratepayer obtained no relief because of this condition. The deputation had the satisfaction of drawing from the Minister an admission that a case had been made out for review. New Zealand is well known as a land of politico-economical experiments; the present system of hospital finance is one of them, and evidently capable of improvement.

### THE GROVE HOSPITAL FOR WOUNDED SOLDIERS.

The Metropolitan Asylums Board have placed at the disposal of the Army Council their Grove Hospital at Tooting Graveney for use as a military hospital for wounded soldiers. The suggestion that the fever hospital might be utilised for the wounded was made by the President of the Local Government Board, who was advised that current statistics indicated that a fever hospital could safely be spared at the present time. The alterations have been speedily made, and the chairman of the Board and the chairman of the hospital committee, who carried out the necessary arrangements, have been thanked by the Army Council and the President of the Local Government Board for the patriotic action of the managers. This hospital contains five hundred beds, and will presumably accommodate that number of wounded soldiers. The Brook, Orchard, and Lower Southern Fever Hospitals are already being used for soldiers. The Belmont Workhouse is also to be converted to the same purpose; it is at present used for epileptics, who will be transferred to Edmonton.

### THE NEW CHAIRMAN OF ADDENBROOKE'S HOSPITAL.

The chairmanship of Addenbrooke's Hospital, Cambridge, rendered vacant by the recent death of Major Pell, who had held office for the past twelve years, has been filled by the unanimous appointment of the Rev. John B. Lock, M.A., Bursar of Caius College, Cambridge, who has been a member of the general committee for the past ten years and vice-chairman for nearly as long. Mr. Lock acted for the late chairman during the latter's enforced absence extending over about 2½ years. In appointing Mr. Lock the general committee paid a very high tribute to the most valuable services he has rendered to the hospital not only as vice-chairman, but as chairman of the house sub-committee and of the building committee.

### A POINT FOR THE PRESIDENT OF THE L.G.B.

Bitter complaint is being made at the delay in the payment by the Local Government Board of the grants in aid to the maternity and infant welfare centres for the year ended March 31. Some of the institutions in various parts of the country are thus placed in a position of much difficulty. The case of Leeds may be taken as typical. There eight clinics and the Wyther Home for sick babies, with a staff consisting of a superintendent and sixteen nurses, are being carried on by the Babies' Welcome Association at a considerable expenditure which the annual subscriptions do not nearly meet. In these circumstances it is not difficult to see that the committee is greatly handicapped by the non-receipt of the Government grant, which will amount to some £500, and is now overdue. No satisfactory explanation as to the delay in paying these grants to infant welfare centres is forthcoming, and it surely should be possible for an arrangement to be made whereby organisations whose work is well known and previously approved might at least receive some instalment of the financial assistance promised, so as to enable them to carry on their work without incurring the incubus of bank overdrafts.

### SPHAGNUM DRESSINGS IN HAMPSHIRE AND BERKSHIRE.

A correspondent who has established a Moss Depôt for Hampshire and Berkshire at Finchampstead writes to inform us of this fact, and also to acknowledge that the whole idea was inspired by the article on "Sphagnum Moss for Dressings" which appeared recently in The Hospital (September 23,

1916; p. 580). Our correspondent has already attained a high level of productivity in her enterprise considering for how short a time it has been established, and she looks forward with confidence to an even larger output in the future. She is also looking well ahead—beyond the present war in fact. She expresses the hope that the War Depôts in general may continue when the war is over to develop English resources and "prevent the previous extravagance of hospitals by inducing them to use home-grown products." We are in complete accord with her that unnecessary extravagance has existed in many hospitals and does still exist, and that every effort should be made to root it out. Over and over again in recent years the gospel of thrift and carefulness for institutions and for workers in them has been preached in these columns. But we question whether after the war the depôts as now conducted will be either practicable or the most economical. That, however, is a matter of opinion; meanwhile, we shall be pleased to give our correspondent's address to anyone who will help her in the sphagnum enterprise or wishes to establish a similar one in another part of the country.

### MUNITION WORKERS AND T.N.T. POISONING.

The two cases of women affected by tri-nitro-toluol poisoning, which accelerated their deaths, brings to light two important factors where women are employed on highly dangerous work. The possibility is that there had been a lack of Government inspection, and in the stress of the times this can be well understood, and the second point is that women, and men too, are apt to become careless and to ignore the safeguards that are provided by their employers for their protection. It is evident that there will have to be a tightening-up of the regulations all round to protect the workers even from their own foolishness and carelessness. Many accidents in the workshops are caused by the carelessness of the employees themselves, who have often little regard to any preventive measure that may be provided for their safety. A careful analysis of the many claims made by workers for compensation under the Workmen's Compensation Act shows that this is frequently the case. Acts of Parliament have been repeatedly passed for the protection of the workers; however, it is one thing to take a horse to the water, but quite another to make him drink. The jury in one of these cases added a rider to their verdict that in future all persons engaged in munition works, whether directly under the Government or not, should be bound by all the regulations referring to preventive measures. It remains to be seen whether this will be acted upon.

### PETTY PATRONAGE IN ITS LAST DITCH.

The system by which deserving candidates are admitted to the benefits of a charity by securing a majority of the subscribers' votes is admittedly imperfect and open to injustice, since more perhaps depends upon the success of a canvass than on the needs or deserts of a particular case. Much criticised in theory the plan survives because the income of institutions which long ago adopted the system depends, or is supposed to depend, on the patronage accorded by it to subscribers. The difficulty experienced in obtaining a large number of votes is so widely felt that devices have to be employed to encourage canvassers. A recent example is one employed in connection with the Royal Hospital for Incurables, Putney Heath. Canvassers are reminded in a brief circular that since "votes for one election are available at the rate of four per guinea, if it is not possible to secure as many votes as interested persons desire, it is sometimes possible to raise money to purchase votes." For this purpose the holding of a tea is suggested to which friends are invited to come, bringing some useful gift with them, carefully marked with its price, and everyone who attends the tea is expected to purchase one article. By this means a sum of five or ten guineas should be raised for the purchase of votes, if such a tea is called "An Allies' Tea"! That the voting system should end in the purchase of votes, though financially advantageous to the institution, is the *reductio ad absurdum* of patronage and voting. We quote the above device as sufficient to condemn that system, while not forgetting that it is the business of hospital officers to maintain their institution under whatever its constitution may lawfully be. It is not their fault if the system is a bad one, but the authorities to whom they are responsible should never be allowed to forget that petty patronage is the enemy of charity, and that it is quite time for the voting system to go.

### THE INITIALS ON THE WALL.

An extension of an old device for raising money is one employed for the benefit of the new Children's Hospital, Birmingham, which at present, owing to financial and industrial difficulties, is only partly finished. A Brick League has been formed—to provide the hospital with linen! But in addition to this object, which necessitates the raising of £150 a year, a further sum of £500 has been raised, presumably for the building fund. We say presumably, since the attraction offered to subscribers is the right to buy a brick already in the building and to have the donor's initials carved upon it. Since £500 has been raised in this manner, the plan seems to be popular. We cannot recall another instance of a hospital raising subscriptions by allowing its structure to be cut in this fashion, though the nominal giving of bricks is nothing new.

### HUMAN TEMPERAMENT.

On another page of this issue (p. 49) will be found the concluding article of a series of studies in Human Temperament. It gives us pleasure to announce that arrangements have been made for the republication of this series, which has attracted widespread interest and attention, in collected form, by The Scientific Press, 28 Southampton Street, Strand, W.C.; and we feel sure that our readers will be glad of the opportunity of reperusing it. The proper study of mankind, as we were told two centuries ago, is man; and the saying is as true now as it was then. All really penetrating analyses of human character, whether exhibited in the guise of fiction or in the form of essays, are true not solely for a day or a year, but for long periods of time; for the sufficient reason that human nature changes very little and very slowly with the passage of centuries. The "Book of Snobs," to look back no further than sixty or seventy years, is as faithful a reflection of British types of character now as it was when it was written. The studies in Temperament which have been appearing in these columns have a slightly different aim, and illustrate a slightly different viewpoint from Thackeray's classic; but in essentials they hold the mirror up to Nature in very much the same way, and they contain lessons for each and all of us which are of very much the same kind. No one can read these studies without uncomfortable qualms of self-examination as he recognises some of those failings which all of us possess; and it is good for any man to see himself, not only as he knows himself to be, but as others may be seeing him—especially if they are as acute observers as the distinguished author of the series which is now shortly to be republished.

### A BELATED APPEAL.

On Wednesday of last week, October 11, the Lord Mayor wrote us, in his capacity of President of the Hospital Saturday Fund, a letter of appeal for support for that excellent charity. We should have been glad to publish this letter if by so doing it had been possible to assist in the slightest way the prosperity of the Saturday Fund; but as it arrived a day or two after our last issue went to press, and only a few hours before "Hospital Saturday" itself, October 14, this was, of course, impossible. With all respect to the Lord Mayor, whose heavy duties and preoccupations naturally prevent his personal supervision of details such as this, we do seriously suggest that in this matter there was room for a better system in dealing with the press. For the daily papers, whose issue of October 13 goes to press in the small hours of the morning of that date, the arrival of such an appeal on October 12 is well timed; though even then the unreliability of the post just now is such that a few hours' margin is a precaution worth taking. But for papers published weekly, a good deal longer notice is essential if "copy" is to be printed just when it is topical and interesting. The matter is of small intrinsic importance, but nevertheless one which should not be neglected.

### THE GREAT NORTHERN CENTRAL HOSPITAL.

The meeting held at the Mansion House on Wednesday to commemorate the Diamond Jubilee of the hospital attracted a large attendance of its friends. The Lord Mayor, who presided, gave a short history of the hospital, which started with the modest establishment of sixteen beds in 1856; it was not until 1885 that the present well-known building in Holloway Road was erected to serve the needs of a population of nearly a million. In this, the Diamond Jubilee year, there are 365 beds available, of which 230 have been set apart for wounded soldiers. The accommodation includes seventeen private wards for paying patients, and is thus in keeping with modern hospital ideas. Last year 2,907 in-patients and 23,115 out-patients were treated, and on an average four operations a day were performed in the operating theatres. When one remembers the busy and populous nature of the districts served it is not surprising to learn that the figures quoted include 10,812 accident cases. The only certain income of the hospital is approximately £1,200 a year, but at present some £25,000 annually is required for maintenance alone. With such a budget the estimated deficit for 1916, £8,000, seems quite insignificant.

### THE BISHOP AND THE WOUNDED.

Such work as that done by the Great Northern Central Hospital deservedly attracts the sympathy and help of well-known men. The Bishop of London, another of the speakers at the Mansion House meeting, expressed the hope that the voluntary hospitals of London would always remain voluntary. His Lordship paid a tribute to the way in which convoys of wounded were dealt with by those in charge of the Ambulance Service and of the reception of patients at the hospitals, calling attention to the short time which elapsed before the men, pain-worn and weary, were clean and comparatively comfortable. The Bishop had always regarded hospitals as great purifying and uplifting agencies, teaching the spirit of self-abnegation and selflessness. The Right Hon. H. J. Tennant, Secretary for Scotland, deputy-chairman of the board of management for the hospitals, spoke from personal knowledge of the inner working of the institution, and his advocacy, added to that of Sir James Crichton Browne and others, has given a powerful impetus to the appeal on the occasion of the hospital Jubilee, which thus commences under the happiest auspices.

### THIS WEEK'S DRUG MARKET.

The steadier tone in the drug market which has been noticeable during the past few weeks is maintained, notwithstanding that business on the whole is quiet. Of course, when one speaks of business as being quiet one means business in the ordinary way; the constant steady buying on the part of the Medical Services of our own and our Allies' armies accounts for a very considerable volume of traffic in drugs which more than makes up for the dulness of the market generally. Price changes of importance have been few. The firmer tone in bromides is maintained. Quinine is in very slow demand, the price of Continental brands still tending downwards. The market for camphor is quieter, but the price is unchanged. The advanced value of opium is well maintained, and a rise in quotations for morphine would not come altogether as a surprise. Saffron is inclined upwards in price. As a result of a considerable demand for potassium permanganate the price has advanced; the output of the British make of this product appears to be increasing. Phenacetin and barbitone continue scarce, but the synthetic drugs generally are practically without noteworthy change in price.

